# Predicting risk of cardiovascular events 1 to 3 years post‐myocardial infarction using a global registry

**DOI:** 10.1002/clc.23283

**Published:** 2019-11-12

**Authors:** Stuart J. Pocock, David Brieger, John Gregson, Ji Y. Chen, Mauricio G. Cohen, Shaun G. Goodman, Christopher B. Granger, Richard Grieve, Jose C. Nicolau, Tabassome Simon, Dirk Westermann, Satoshi Yasuda, Katarina Hedman, Kirsten L. Rennie, Karolina Andersson Sundell, for the TIGRIS Study Investigators

**Affiliations:** ^1^ Department of Medical Statistics, London School of Hygiene and Tropical Medicine London UK; ^2^ Division of Cardiology, Concord Hospital and University of Sydney Sydney Australia; ^3^ Department of Cardiology, Guangdong General Hospital, Provincial Key Laboratory of Coronary Disease Guangzhou China; ^4^ Cardiovascular Division, University of Miami Miller School of Medicine Miami Florida; ^5^ Already given, Terrence Donnelly Heart Centre, St Michael's Hospital, University of Toronto Toronto Canada; ^6^ Cardiac Intensive Care Unit, Duke Clinical Research Institute, Duke University Medical Center Durham North Carolina; ^7^ Instituto do Coração (InCor), Hospital das Clínicas HCFMUSP, Faculdade de Medicina, Universidade de São Paulo São Paulo SP Brazil; ^8^ Assistance Publique‐Hopitaux de Paris (APHP) Department of Clinical Pharmacology and Clinical Research Platform of East of Paris Paris France; ^9^ Department of Pharmacology, Sorbonne‐Université (UPMC‐Paris 06) Paris France; ^10^ Department of General and Interventional Cardiology University Heart Center Eppendorf Hamburg Germany; ^11^ German Center for Cardiovascular Research (DZHK) Partner site Hamburg/Lübeck/Kiel Hamburg Germany; ^12^ Department of Cardiovascular Medicine, National Cerebral and Cardiovascular Center Osaka Japan; ^13^ Global Medical Affairs Cardiovascular, Renal and Metabolic, AstraZeneca Gothenburg Sweden; ^14^ Oxon Epidemiology (UK) London UK

**Keywords:** cardiac risk factors and prevention, coronary artery disease, myocardial infarction

## Abstract

**Background:**

Risk prediction tools are lacking for patients with stable disease some years after myocardial infarction (MI).

**Hypothesis:**

A practical long‐term cardiovascular risk index can be developed.

**Methods:**

The long‐Term rIsk, Clinical manaGement and healthcare Resource utilization of stable coronary artery dISease in post‐myocardial infarction patients prospective global registry enrolled patients 1 to 3 years post‐MI (369 centers; 25 countries), all with ≥1 risk factor (age ≥65 years, diabetes mellitus requiring medication, second prior MI, multivessel coronary artery disease, or chronic non‐end‐stage kidney disease [CKD]). Self‐reported health was assessed with EuroQoL‐5 dimensions. Multivariable Poisson regression models were used to determine key predictors of the primary composite outcome (MI, unstable angina with urgent revascularization [UA], stroke, or all‐cause death) over 2 years.

**Results:**

The primary outcome occurred in 621 (6.9%) of 9027 eligible patients: death 295 (3.3%), MI 195 (2.2%), UA 103 (1.1%), and stroke 58 (0.6%). All events accrued linearly. In a multivariable model, 11 significant predictors of primary outcome (age ≥65 years, diabetes, second prior MI, CKD, history of major bleed, peripheral arterial disease, heart failure, cardiovascular hospitalization (prior 6 months), medical management (index MI), on diuretic, and poor self‐reported health) were identified and combined into a user‐friendly risk index. Compared with lowest‐risk patients, those in the top 16% had a rate ratio of 6.9 for the primary composite, and 18.7 for all‐cause death (overall *c*‐statistic; 0.686, and 0.768, respectively). External validation was performed using the Australian Cooperative National Registry of Acute Coronary Care, Guideline Adherence and Clinical Events registry (*c*‐statistic; 0.748, and 0.849, respectively).

**Conclusions:**

In patients >1‐year post‐MI, recurrent cardiovascular events and deaths accrue linearly. A simple risk index can stratify patients, potentially helping to guide management.

## 1 INTRODUCTION

Many low‐risk patients with stable coronary disease following myocardial infarction (MI) continue to live unencumbered by recurrent cardiovascular events, while high‐risk patients may benefit from more intensive medical therapy.

Established risk scores exist for patients with MI both from admission^1^, ^2^ and from discharge,^3^, ^4^ but risk prediction tools for longer‐term management of patients with stable disease following MI are lacking. The long‐Term rIsk, clinical manaGement and healthcare Resource utilization of stable coronary artery dISease in post‐myocardial infarction patients (TIGRIS) prospective registry evaluated a large representative group of patients recruited 1 to 3 years post‐MI.^5^

Given the recent availability of new therapies that can improve outcomes in these patients,^6^, ^7^, ^8^ there is a need to understand the drivers of risk in this cohort so as to identify those at greatest absolute risk who are likely to sustain the greatest absolute benefit.

This article focuses on incidence of the composite primary endpoint (MI, unstable angina requiring revascularization, stroke, and all‐cause death) over 2 years, and characterizes any influence by baseline patient factors. Our goal was to develop a user‐friendly risk index incorporating readily available items, see how strongly it facilitated risk discrimination for the primary endpoint and for all‐cause death, and validate against an external population. Finally, we discuss how this risk index may be used in patients with stable coronary disease to determine those with relatively good prognosis and those at higher risk who may benefit from more intensive management.

## 2 HYPOTHESIS

That a practical cardiovascular risk index could be developed using data from a global registry of patients >1 year post‐MI, followed up for 2 years.

## 3 METHODS

### 3.1 Study design and patients

TIGRIS is a prospective, global registry of patients enrolled 1 to 3 years post‐MI in 25 countries in Europe, North America, Latin America, Asia, and Australia, followed for 2 years. The study design and patient characteristics have been described.^5^, ^9^ Eligible patients had at least one of the following: age ≥65 years, diabetes mellitus requiring medication, a second prior MI, angiographic evidence of multivessel disease, and chronic non‐end‐stage renal dysfunction.

TIGRIS was performed in accordance with ethical principles that are consistent with the Declaration of Helsinki, the International Conference on Harmonization Good Clinical Practice Guidelines, and applicable legislation on nonintervention studies. All patients provided written informed consent. The study protocol and informed consent was reviewed and approved by the corresponding health authorities and ethics boards for all participating study sites. This includes China HGR approval of inclusion of 750 Chinese patients. The study was registered at https://ClinicalTrials.gov (clinical trial identifier NCT01866904).

Eligible patients who had survived an acute coronary syndrome (ACS) were enrolled at discharge from participating centers and subsequently contacted every 6 months by phone or at study sites to ascertain outcome events and changes in medications. All outcome events reported by patients and relatives (eg, hospitalizations, cardiovascular events, deaths) were confirmed by the study sites.

### 3.2 Risk index development

The predefined primary composite endpoint of MI, unstable angina with urgent revascularization (UA), stroke, and all‐cause death showed a linear accumulation of events over time.^10^ Hence, incidence of the primary outcome by baseline variables was reported as rate per 100 patient‐years. Poisson regression models simultaneously estimated the association of several baseline variables with risk of events expressed as incidence rate ratios (IRRs) and 95% confidence intervals.

We used forward stepwise variable selection to derive a preliminary multivariable predictive model for risk of the primary outcome. The five high‐risk eligibility criteria were forced into this model, as were sex and geographic region. All other variables needed to achieve *P* < .05 for inclusion. A final condensed Poisson model for the primary endpoint was obtained by only including variables significant at *P* < .01, and by modeling age (<65 and ≥65 years) and diabetes as binary variables.

To assess the risk impact of self‐reported health, the 3‐level EuroQoL‐5 dimensions (EQ‐5D‐3L) survey instrument^11^ was used. Patients graded five dimensions (mobility, self‐care, usual activities, pain/discomfort, and anxiety/depression) as no, moderate, or severe problem scoring each as 0, 1, or 2 points, respectively, and summing to yield a simple overall score (range 0‐10). Recommended EQ‐5D scorings such as UK‐weighted index^12^ are complex and impractical for clinical use.

The final predictive model was converted into an integer risk index: variables with rate ratios ranging from 1.33 (congestive heart failure) to 1.69 (prior major bleed) were each assigned 1 point, while the strongest predictor EQ‐5D‐3L overall score ≥4 with rate ratio 2.06 was assigned 2 points.

This risk index was formed into six ordered categories from 0 points to ≥5 points. Risk discrimination was quantified using incidence rates, IRRs using 0 points as reference, and Kaplan‐Meier plots over 2 years. Harrell's *c*‐statistic summarized the strength of discrimination.^13^

### 3.3 External validation

External validation of the risk index used the Australian Cooperative National Registry of Acute Coronary Care, Guideline Adherence and Clinical Events (CONCORDANCE), which included 4672 post‐MI patients seen 6 months post‐discharge.^14^ Occurrences of the primary composite outcome and death were documented at 6 months (n = 3197) or 18 months (n = 1451). Precise event dates were unavailable with logistic regression used to predict outcomes, adjusting for duration of follow‐up. EQ‐5D was missing in 1588 patients in CONCORDANCE. We therefore used multiple imputation using five imputed datasets, combining results using Rubin's rules^15^; predictor variables were the primary outcome, death, and other risk model variables.

All statistical analyses used Stata version 15.1.

## 4 RESULTS

### 4.1 Patient characteristics

From June 2013 to November 2014, 9225 patients were enrolled; median 1.8 years post‐MI with at least one pre‐defined risk factor. From these, 9027 (97.9%) had baseline and follow‐up data: age ≥65 years (5626 [62.3%] patients), diabetes mellitus requiring medication (3018 [33.4%]), a second prior MI (924 [10.2%]), multivessel disease (5941 [65.8%]), and chronic non‐end‐stage renal dysfunction (691 [7.7%]). Patient characteristics recorded at enrollment and numbers enrolled by country are provided in Tables S1 and S2.

### 4.2 Primary outcome

The primary outcome occurred in 621 (6.9%) from 9027 patients over 2 years, with all‐cause death in 295 (3.3%), MI in 195 (2.2%), unstable angina requiring revascularization in 103 (1.1%), and stroke in 58 (0.6%) patients.

For the primary outcome and components, a steady linear accumulation of events occurred over time.^10^

### 4.3 Identifying predictors of risk

Univariate associations of patient characteristics to the primary outcome incidence rate are provided in Table S1, with IRRs shown both unadjusted and adjusted for the five eligibility criteria, region, and country. Unadjusted incidence rates by region and country are shown in Table S2.

To identify which variables remained statistically significant independent risk predictors in multivariable analyses, forward stepwise variable selection was used to derive a preliminary predictive model for risk of the primary outcome (Table 1). The influence of age is effectively summarized by elevated risk for age ≥65 years. Diabetes is an important risk predictor, particularly for insulin‐treated patients, as are having a second prior MI, a prior major bleed, peripheral arterial disease, and prior heart failure. Also, cardiovascular hospitalization in the last 6 months, medical management only for the index MI, and diuretic therapy at enrollment also carried elevated risk.

**Table 1:** Multivariable Poisson regression model for the risk of the primary composite outcome (based on forward stepwise variable selection^a^)

Variable at enrollment	Rate ratio (95% CI)	*P* value
Age, years		
<55	Reference group	.030
55‐59	1.02 (0.68, 1.53)	
60‐64	1.13 (0.77, 1.67)	
65‐69	1.49 (1.03, 2.14)	
70‐74	1.40 (0.97, 2.03)	
75‐79	1.54 (1.05, 2.25)	
80+	1.58 (1.05, 2.38)	
Female	0.87 (0.72, 1.05)	.15
Category of diabetes		
No diabetes	Reference group	<.001
Non‐insulin‐treated diabetes	1.30 (1.07, 1.57)	
Insulin‐treated diabetes	1.71 (1.36, 2.15)	
Second prior MI	1.45 (1.18, 1.80)	<.001
Multi‐vessel disease	1.14 (0.95, 1.37)	.15
Chronic kidney disease	1.53 (1.22, 1.92)	<.001
Major bleed	1.67 (1.21, 2.32)	.002
Peripheral arterial disease	1.45 (1.14, 1.84)	.002
Congestive heart failure	1.30 (1.05, 1.60)	.015
Cardiovascular event in past 6 months	1.39 (1.05, 1.84)	.021
On diuretics at enrollment	1.57 (1.31, 1.88)	<.001
Type of anti‐thrombotic medication		
SAPT	Reference group	.025
No APT	1.30 (0.97, 1.75)	
DAPT	1.25 (1.04, 1.52)	
Type of management of index MI		
PCI	Reference group	<.001
CABG	0.79 (0.56, 1.11)	
Medical only	1.58 (1.28, 1.95)	
EQ‐5D overall score (0–10)		
0	Reference group	<.001
1	1.14 (0.90, 1.43)	
2	1.30 (1.01, 1.67)	
3	1.61 (1.23, 2.12)	
4+	2.25 (1.76, 2.89)	
Region		
Asia and Australia	Reference group	.72
Europe	0.99 (0.70, 1.39)	
North America	1.07 (0.64, 1.79)	
Latin America	1.22 (0.80, 1.87)	

Abbreviations: APT, antiplatelet therapy; CABG, coronary artery bypass graft; CI, confidence interval; DAPT, dual antiplatelet therapy; EQ‐5D, EuroQol‐5 dimensions; MI, myocardial infarction; PCI, percutaneous coronary intervention; SAPT, single antiplatelet therapy.

a Sex, region, and the five eligibility criteria were forced into the model.

The EQ‐5D findings indicate the prognostic importance of patient‐reported heath status with each constituent item showing increased univariate trends of risk from no‐to‐some‐to‐severe problems (Table S1). Their sum yielded an overall ED‐5D‐3L score ranging from 0 (no problems on all five items) to 10 (severe problems on all five items). An overall score of 3 has a significantly elevated risk, which increased further for a score of ≥4 (Table 1). The EQ‐5D visual analog score did not independently predict risk.

Sex, multivessel disease, and region were not statistically significant independent predictors. Patients on single antiplatelet therapy at enrollment appeared to have a lower risk. The underlying selection processes are unknown so this factor was not considered further. Variables expected to predict risk (eg, low blood pressure, elevated heart rate) were not independent predictors.

### 4.4 Final predictive model

A final refined predictive model for the primary outcome is shown in Table 2, including 11 yes/no items independently contributing highly significant IRRs ranging from 1.33 to 1.69. For EQ‐5D overall score, values of 3 and ≥4 contributed IRRs of 1.47 and 2.06, respectively.

**Table 2:** A refined predictive model for risk of the primary composite outcome and simplified scoring for the risk index

Variable	Percentage of TIGRIS patients affected^a^	Rate ratio (95% CI)	*P* value	Contribution to risk index^b^
Age ≥ 65 years	62.3	1.34 (1.12, 1.60)	.001	1
Diabetes	33.4	1.42 (1.20, 1.67)	<.001	1
Second prior MI	10.2	1.52 (1.24, 1.88)	<.001	1
Chronic kidney disease	7.7	1.61 (1.29, 2.02)	<.001	1
Heart failure	11.4	1.33 (1.08, 1.64)	.008	1
Peripheral arterial disease	6.7	1.52 (1.20, 1.93)	<.001	1
Cardiovascular event in past 6 months	4.8	1.46 (1.11, 1.93)	.008	1
Prior major bleed	2.8	1.69 (1.22, 2.34)	.002	1
Medical management only of index event	11.9	1.62 (1.33, 1.99)	<.001	1
On diuretic at enrollment	25.1	1.62 (1.35, 1.93)	<.001	1
EQ‐5D overall score^c^ of 3	7.8	1.47 (1.15, 1.88)	<.001	1
EQ‐5D overall score^c^ ≥ 4	9.0	2.06 (1.67, 2.55)	<.001	2

Abbreviations: CI, confidence interval; EQ‐5D, EuroQol‐5 dimensions; MI, myocardial infarction; TIGRIS, long‐Term rIsk, Clinical manaGement and healthcare Resource utilization of stable coronary artery dISease in post‐myocardial infarction patients.

a Among 8978 patients with complete information on covariates in the risk index.

b The rate ratio for EQ‐5D overall score ≥4 was 2.06 while the rate ratio for all other items ranged from 1.33 to 1.69. In the interests of practical simplicity, 2 points have been assigned to the former and 1 point to each of the others.

c The EQ‐5D grades five dimensions (mobility, self‐care, usual activities, pain/discomfort, anxiety/depression) as no, moderate, or severe problem. Scoring each as 0, 1, or 2 points, respectively, and adding these up yields an overall score ranging from 0‐10. A score of 3 points means a patient had either: (a) three dimensions with moderate problem or (b) one dimension with moderate problem and one dimension with severe problem. A score of 4 or more points means a patient had at least either: (a) four dimensions with moderate problem; (b) two dimensions with moderate problem and one dimension with severe problem; (c) two dimensions with severe problem.

To make this prediction model more user‐friendly, we propose a risk index for each patient (Table 2). All items contributed 1 point to the risk index, except for EQ‐5D overall score ≥4 (2 points). Distribution of the risk index for 8978 patients with complete information is shown in Figure S1. A value of 0 points occurred in 12% of patients, 1 point was most common (33% of patients), followed by a skewed distribution to a maximum of 10 points in four patients.

**Figure 1:**
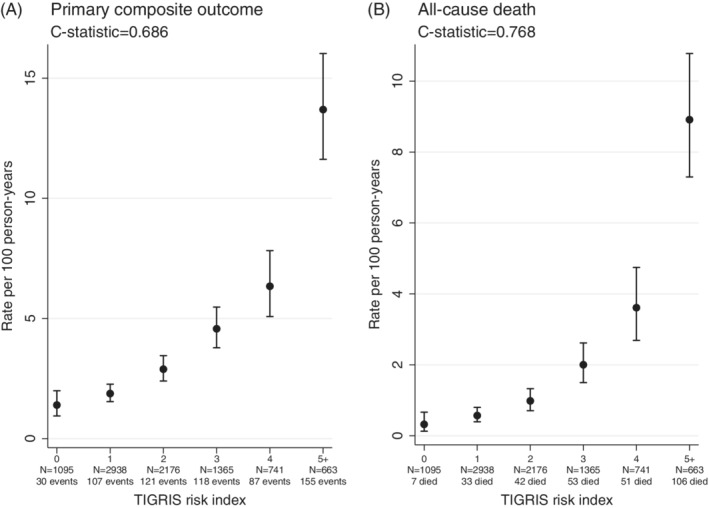
Rate per 100 person‐years by categories of the risk index in the TIGRIS study population for the primary composite outcome (death, myocardial infarction, unstable angina, and stroke) and for all‐cause death. Abbreviation: TIGRIS, long‐Term rIsk, Clinical manaGement and healthcare Resource utilization of stable coronary artery dISease in post‐myocardial infarction patients

**Figure 2:**
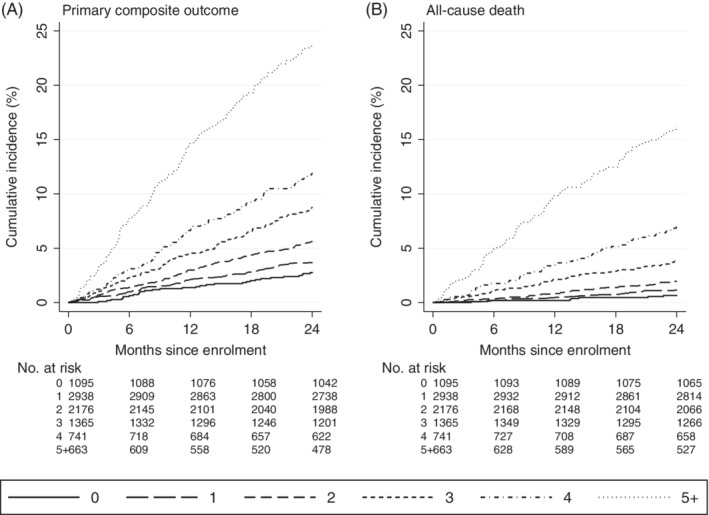
Kaplan–Meier plots of primary composite outcome and all‐cause death, each by six categories of the risk index

Figures 1A and 2A show a marked trend in risk of the primary outcome. Compared to 0 points, IRRs ranged from 1.34 for 1 point to 4.54 and 9.79 for those with 4 and ≥5 points, respectively. To assess goodness of fit of the model, we compared the observed and predicted rates for the primary outcome across risk index categories (see Figure S2). Figure 2A shows cumulative incidence of the primary outcome by categories of the risk index, revealing marked separation in risk. The 2‐year cumulative incidence for the primary outcome ranged from 2.8% for 0 points up to 11.9% and 23.7% for 4 and ≥5 points, respectively. For all‐cause death, compared to patients with 0 points, there is an even steeper risk gradient (IRR) ranging from 1.77 for 1 point to 11.19 and 27.61 for 4 and ≥5 points, respectively (Figures 1 and 2B). *c*‐Statistics for the risk index are 0.686 for the primary outcome and 0.768 for all‐cause death.

### 4.5 External validation

**Figure 3:**
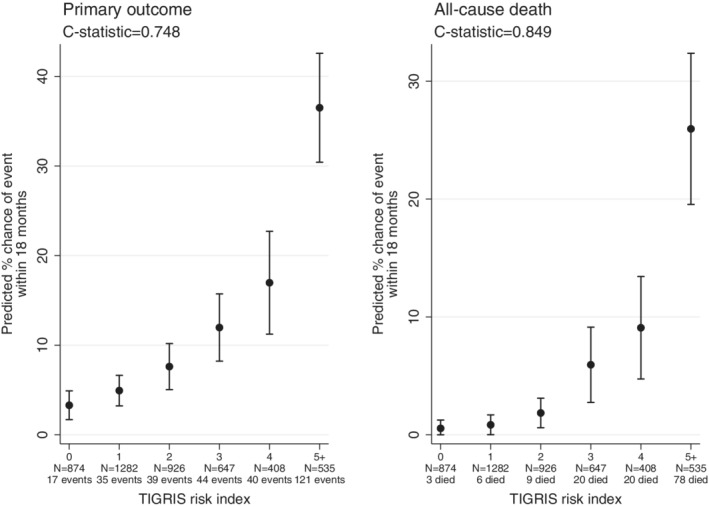
Predicted percentage chance of an event occurring within 18 months of enrollment in the CONCORDANCE study population for the primary composite outcome (death, myocardial infarction, unstable angina, and stroke) and for all‐cause death. Abbreviations: CONCORDANCE, Australian Cooperative National Registry of Acute Coronary Care, Guideline Adherence and Clinical Events, TIGRIS, long‐Term rIsk, Clinical manaGement and healthcare Resource utilization of stable coronary artery dISease in post‐myocardial infarction patients

External validation of this risk index was explored comparing TIGRIS with the CONCORDANCE registry,^14^ comprising 4672 Australian patients recruited 6‐months post‐MI. While primary outcome incidence patterns are broadly similar, some differences exist regarding baseline variables (Table S3). When applying the TIGRIS risk index to CONCORDANCE, we observed similar, markedly steep gradients for both the primary composite outcome and all‐cause death (Figure 3). *c*‐Statistics for the primary outcome (*c* = 0.748) and all‐cause death (*c* = 0.849) are somewhat greater than in the TIGRIS population. It is difficult to attribute a definitive cause for this. Notably, associations for age, congestive heart failure, and cardiovascular events are stronger in CONCORDANCE than TIGRIS. It follows, therefore, that the presence of one of these characteristics will have a larger impact on underlying risk in the former, making it easier to discriminate patients (see Table S4). In addition, risk factors strongly associated with risk in CONCORDANCE are also generally more common (medical management, CV event in the prior 6 months). Thus, in general, patients are more diverse in terms of the risk factors they present with, again facilitating easier discrimination.

## 5 DISCUSSION

In a global representative sample of patients 1 to 3 years post‐MI with ≥1 risk factor, we have quantified what determines the truly high‐risk patient with stable coronary disease.

The 11 highly significant independent predictors, including patient self‐reported health status, are readily available in routine clinical practice. Combining them into an integer risk index provides an easy‐to‐use method of assessing individual post‐MI risk of a major cardiovascular event or death over 2 years.

There is marked variation in individual patient risk, ranging from 12.2% of patients with risk index 0 points (primary outcome incidence rate of 1.4 per 100 patient‐years) to 7.4% of patients with ≥5 points (13.7 per 100 patient‐years): IRR 9.8. The risk gradient for primary outcome is steep, whereby each added point contributed ≥30% extra cumulative risk. For all‐cause death, the gradient becomes steeper with ≥75% extra cumulative mortality risk per point.

Independent contribution of increasing age is best captured by age over 65 years with increased risk represented by increased susceptibility to comorbidities and poorer quality of life included separately in the risk index.

Other concomitant conditions (diabetes, chronic kidney disease, heart failure, peripheral arterial disease and >1 MI) all contribute to increased risk. These likely reflect the greater burden of vascular disease in these patients. Having their MI medically managed only, and history of major bleeding also contributed to increased risk, possibly reflecting failure to tolerate or be offered prognostically important therapies. All of these factors have contributed to other risk scores in stable and unstable populations.^3^, ^4^, ^16^, ^17^, ^18^

A more innovative contributor to our risk index is patient self‐reported health status, using a simple overall score derived from the EQ‐5D‐3L. Patients with poor self‐reported health (3 points) had around a 50% increase in cardiovascular incidence rate, while those with very poor self‐reported health (≥4 points) had around a doubling of incidence rate (Table 2). This simple patient rating of health status was the strongest contributor to risk. The EQ‐5D instrument is also reported as a strong predictor of mortality after discharge post‐MI.^3^, ^4^ The reasons for this are speculative; in some patients the EQ‐5D may be unmasking undetected depression, a known adverse marker of poor prognosis,^19^ or it may be that patients with poorer self‐reported quality of life are less likely to adhere to prescribed medications, or to attend cardiac rehabilitation, behaviors which have both been shown to adversely affect long‐term outcomes^20^, ^21^

We also studied the UK‐weighted index score for the EQ‐5D‐3L, revealing results comparable to our easier‐to‐use overall score.

For external validation, we used the CONCORDANCE registry^14^ as it included all items in our risk index, and data on our primary outcome. This Australian population had follow‐up starting 6 months after MI, so not a perfect match to our TIGRIS population. Nevertheless, the risk index achieves a broadly similar extent of risk discrimination for both the primary outcome and death. It is of considerable practical value to be able to identify which patients with stable coronary disease are at high risk of cardiovascular events and death. For instance, there are important risk reductions with new therapies for LDL‐lowering^6^ and relating to antithrombotic therapy.^7^, ^8^ Identifying the spectrum of risk in stable patients eligible for such treatments will identify those high‐risk patients for whom the absolute reduction will be greatest.

Reported risk models for patients with stable coronary disease post‐MI are sparse. Closest is perhaps the Reduction of Atherothrombosis for Continued Health (REACH) Registry,^22^ which included a mixed population comprising coronary heart disease, cerebrovascular disease, and peripheral vascular disease. Their model for predicting cardiovascular event risk over 20 months included 11 items (six common to our risk index) but is dominated by increasing risk with age. Applied to TIGRIS, the REACH score showed weaker discrimination: *c*‐statistic 0.62 compared with 0.69 for ours.

Based on the Thrombin Receptor Antagonist in Secondary Prevention of Atherothrombotic Ischemic Events (TRA 2°P‐TIMI 50) trial,^23^ a multivariable risk model was derived for cardiovascular death, MI, and ischemic stroke over a median 2.5 years in 8598 placebo‐treated patients recruited 2 weeks to 1‐year post‐MI. Their population is earlier post‐MI, with 45% recruited within 3 months and 74% within 6 months.^24^ Thus, early follow‐up is in the post‐acute phase when mortality is double that at 1‐year post‐MI.^25^ Their risk model contains nine items, three of which (smoking, prior coronary artery bypass graft, and hypertension) were not independent predictors when applied to TIGRIS. Hence, applying the TIMI‐50 risk model to our population showed weaker discrimination: *c*‐statistic 0.63 and 0.70 for the primary outcome and all‐cause death, respectively. However, we acknowledge that the *c*‐statistic for our own risk score may be slightly optimistic, given that it is derived and assessed in the same population.

Battes et al^26^ studied patients in the EURopean trial On reduction of cardiac events with Perindopril in stable coronary Artery disease (EUROPA) database: 65% had a prior MI ≥3 months beforehand. Their risk models, over a median follow‐up of 4.1 years, had 11 predictor variables for cardiovascular death and a composite outcome including MI and cardiac arrest; the *c*‐statistics were 0.73 and 0.63, respectively, a weaker prediction than we found. Rapsomaniki et al^27^ used electronic health records for 102 023 patients with stable coronary disease, 23% with prior MI ≥6 months ago. Their risk models over a mean 4.4 years included 21 predictor variables for all‐cause death and the composite of coronary death and MI; *c*‐statistics were 0.81 and 0.78, respectively. While these showed excellent predictive power, model complexity may limit practical value. Clayton et al^28^ studied 7311 patients with stable angina in A Coronary disease Trial Investigating Outcome with Nifedipine GITS (ACTION); 51% of whom had a prior MI. Their risk model over a mean 4.9 years included 16 predictors for a composite of all‐cause death, MI, and stroke; again, complexity may limit utility.

Regarding our study's limitations, based on 50 candidate predictors, there is risk of “false positives” entering our risk model. However, with *P* < .01 as entry criterion, risk is relatively low. The most novel highly significant predictor is EQ‐5D overall score, while other risk index items are not surprising, having rational explanations based on prior studies. One problem potentially affecting the widespread use of our risk index may be that patients self‐reported health status by EQ‐5D is not routinely collected in most clinical settings. This is an emerging concern given the recognition of the importance of patient reported outcomes as indicators of the quality of care they receive.^29^ In showing that patient reported outcomes makes a clear contribution to their prognosis, we provide additional justification for the importance of collection of data on patient symptom status and encourage a wider appreciation that an assessment of patient self‐perception of their well‐being is an important component of patient care.

While TIGRIS was designed to recruit representative patients in representative centers in each country, we cannot verify a truly generalizable population. Also, TIGRIS recruitment required each patient to have ≥1 of 5 risk criteria, four of which (all except multi‐vessel coronary artery disease) are in the risk model. Thus, in applying our risk index to an unselected population of patients, a higher proportion may be identified as low risk.

We have successful external validation for our risk index using the CONCORDANCE registry and would encourage further validation studies in other relevant populations. While we studied patients recruited 1 to 3 years post‐MI, it would be interesting to see how our risk index performs in a broader class of patients with stable coronary disease.

## 6 CONCLUSION

We have described how risk of subsequent cardiovascular events and death varies substantially among patients 1 to 3 years post‐MI. A risk index based on 11 readily available items enables stratification of such patients into markedly different risk categories. Such knowledge could help identify very high‐risk patients in need of more intensive management.

## 7 DATA SHARING STATEMENT

Baseline patient characteristics and data on long‐term oral antiplatelet use and event rates in the TIGRIS registry have been published elsewhere (all open access).^5^, ^9^, ^10^

## CONFLICTS OF INTERESTS

Richard Grieve declares no potential conflict of interest. Stuart J. Pocock reports research grant support from AstraZeneca. David Brieger reports speaker/consulting honoraria and/or research grant support from Amgen Inc., AstraZeneca, Bayer, Boehringer Ingelheim, Bristol Myers Squibb, Eli Lilly, Merck, Sanofi. John Gregson reports grants from AstraZeneca during the conduct of the study and outside the submitted work. Ji Y. Chen reports research grant support from AstraZeneca; consulting honoraria from MicroPort, APT Medical and JW Medical. Mauricio G. Cohen Speaker/consulting honoraria and/or research grant support from AstraZeneca, Medtronic, Abiomed, Merit Medical. Shaun G. Goodman reports speaker/consulting honoraria and/or research grant support from Amgen, AstraZeneca, Bayer, Boehringer Ingelheim, Bristol Myers Squibb, CSL Behring, Daiichi Sankyo, Eli Lilly, Fenix Group International, Ferring Pharmaceuticals, GlaxoSmithKline, Janssen/Johnson & Johnson, Luitpold Pharmaceuticals, Matrizyme, Merck, Novartis, Pfizer, Regeneron, Sanofi, Servier, Tenax Pharmaceuticals. Christopher B. Granger reports consulting honoraria and/or research grant support from Armetheon, AstraZeneca, Bayer, Boehringer Ingelheim, Bristol‐Myers Squibb, Daiichi Sankyo Eli Lilly, Gilead, Glaxo SmithKline, Hoffmann‐La Roche, Janssen Pharmaceuticals, Metronic, Pfizer, Salix Pharmaceuticals, Sanofi, Takeda, The Medicines Company. Jose C. Nicolau reports speaker/consulting honoraria and/or research grant support from Amgen, AstraZeneca, Bayer, BMS, Boehringer Ingelheim, GSK, Merck, Novartis, Pfizer, Sanofi. Tabassome Simon reports speaker/consulting honoraria and/or research grant support from Astellas, Amgen Inc, AstraZeneca, Bayer, Boehringer Ingelheim, Eli Lilly, GlaxoSmithKline, Merck, Novartis, Pfizer, Sanofi. Dirk Westermann Speaker/consulting honoraria and/or research grant support from AstraZeneca, Bayer, Berlin‐Chemie, Biotronik, Novartis. Satoshi Yasuda reports speaker/consulting honoraria and/or research grant support from Takeda, Daiichi‐Sankyo, AstraZeneca, Boehringer Ingelheim, Bristol‐Myers. Katarina Hedman reports employee of AstraZeneca. Kirsten L. Rennie is an employee of OXON Epidemiology Ltd., and a paid consultant to AstraZeneca in connection with the conduct, data management, and analyses of this study. Karolina A. Sundell is an employee of AstraZeneca.

## Supporting information


**Appendix S1**: Supplementary Material
