# Supplementary material for: Predicting risk of cardiovascular events 1 to 3 years post‐myocardial infarction using a global registry
Source: Clin Cardiol. 2019 Nov 12;43(1):24–32. doi: 10.1002/clc.23283 (PMC6954378; doi:10.1002/clc.23283)
Supplement: Supplementary file 1 — Appendix S1: Supplementary Material [file CLC-43-24-s001.docx]

# Supplementary Material

**eTable 1.** Patient characteristics at enrollment and corresponding incidence of the primary composite outcome

**eTable 2.** Incidence of the primary composite endpoint by region and by country

**eTable 3.** Comparison of TIGRIS and CONCORDANCE study populations

**eTable 4.** Comparison of risk models for the primary CV outcome in TIGRIS and CONCORDANCE

**eFigure 1.** Distribution of risk index in the TIGRIS study population

**eFigure 2.** Model observed and predicted risk for the CV composite

eTable 1. Patient characteristics at enrolment and corresponding incidence of the primary composite outcome^a^

|  | Patients | No. with primary outcome (rate per 100 person-years) | Crude rate ratio (95% CI) | Adjusted rate ratio* (95% CI) |
| --- | --- | --- | --- | --- |
| All patients | 9027 | 621 (3.6) |  |  |
| **Age (years)** | | | | |
| <55 | 814 (9.0%) | 38 (2.4) | 1.00 (reference) | 1.00 (reference) |
| 55-59 | 1206 (13.4%) | 62 (2.7) | 1.12 (0.75 to 1.67) | 1.07 (0.71 to 1.60) |
| 60-64 | 1378 (15.3%) | 81 (3.1) | 1.27 (0.87 to 1.87) | 1.18 (0.80 to 1.74) |
| 65-69 | 2080 (23.0%) | 141 (3.5) | 1.47 (1.03 to 2.11) | 1.53 (1.07 to 2.20) |
| 70-74 | 1775 (19.7%) | 130 (3.8) | 1.59 (1.11 to 2.28) | 1.58 (1.09 to 2.28) |
| 75-79 | 1125 (12.5%) | 99 (4.7) | 1.94 (1.34 to 2.83) | 1.83 (1.25 to 2.68) |
| 80+ | 649 (7.2%) | 70 (5.8) | 2.43 (1.64 to 3.60) | 2.19 (1.46 to 3.28) |
| **Body mass index** | | | | |
| Underweight (<20kg/m2) | 267 (3.0%) | 25 (5.0) | 1.48 (0.97 to 2.25) | 1.38 (0.90 to 2.11) |
| Normal weight (20-24.9 kg/m2) | 2596 (29.6%) | 168 (3.4) | 1.00 (reference) | 1.00 (reference) |
| Overweight (25-29.9 kg/m2) | 3806 (43.4%) | 233 (3.2) | 0.95 (0.78 to 1.15) | 0.87 (0.71 to 1.06) |
| Obese (30+ kg/m2) | 2103 (24.0%) | 176 (4.4) | 1.31 (1.06 to 1.62) | 1.09 (0.87 to 1.37) |
| **Systolic blood pressure (mmHg)** | | | | |
| <104 mmHg | 410 (4.6%) | 37 (4.8) | 1.34 (0.93 to 1.92) | 1.31 (0.91 to 1.88) |
| 105-114mmHg | 966 (10.9%) | 71 (3.9) | 1.08 (0.81 to 1.44) | 1.04 (0.78 to 1.38) |
| 115-124 mmHg | 2080 (23.6%) | 142 (3.6) | 1.00 (reference) | 1.00 (reference) |
| 125-134 mmHg | 1844 (20.9%) | 109 (3.1) | 0.87 (0.67 to 1.11) | 0.86 (0.67 to 1.10) |
| 135-144 mmHg | 1806 (20.5%) | 115 (3.3) | 0.93 (0.73 to 1.19) | 0.83 (0.65 to 1.07) |
| 145-155 mmHg | 792 (9.0%) | 58 (3.9) | 1.08 (0.80 to 1.46) | 0.91 (0.67 to 1.24) |
| 155mmHg+ | 927 (10.5%) | 65 (3.7) | 1.03 (0.77 to 1.38) | 0.82 (0.61 to 1.11) |
| **Diastolic blood pressure (mmHg)** | | | | |
| <65 mmHg | 1248 (14.2%) | 111 (4.7) | 1.46 (1.15 to 1.84) | 1.26 (0.99 to 1.60) |
| 65-74 mmHg | 2784 (31.6%) | 202 (3.8) | 1.17 (0.96 to 1.42) | 1.11 (0.91 to 1.35) |
| 75-84 mmHg | 3162 (35.9%) | 197 (3.3) | 1.00 (reference) | 1.00 (reference) |
| 85-94 mmHg | 1272 (14.4%) | 65 (2.7) | 0.82 (0.62 to 1.08) | 0.80 (0.61 to 1.06) |
| 95 mmHg+ | 351 (4.0%) | 21 (3.1) | 0.95 (0.61 to 1.50) | 0.93 (0.59 to 1.46) |
| **Heart rate (bpm)** | | | | |
| <60 bpm | 1613 (18.5%) | 97 (3.1) | 1.00 (reference) | 1.00 (reference) |
| 60-69 bpm | 3249 (37.3%) | 204 (3.3) | 1.05 (0.82 to 1.33) | 1.01 (0.79 to 1.29) |
| 70-79 bpm | 2500 (28.7%) | 174 (3.7) | 1.17 (0.91 to 1.49) | 1.17 (0.91 to 1.52) |
| 80-89 bpm | 1010 (11.6%) | 87 (4.6) | 1.45 (1.09 to 1.94) | 1.47 (1.10 to 1.98) |
| 90 bpm+ | 333 (3.8%) | 28 (4.4) | 1.41 (0.93 to 2.15) | 1.41 (0.92 to 2.15) |
| **Sex** | | | | |
| Male | 6866 (76.1%) | 462 (3.5) | 1.00 (reference) | 1.00 (reference) |
| Female | 2161 (23.9%) | 159 (3.9) | 1.10 (0.92 to 1.32) | 1.01 (0.84 to 1.21) |
| **Smoking status** | | | | |
| Never Smoked | 3398 (37.7%) | 209 (3.2) | 1.00 (reference) | 1.00 (reference) |
| Former Smoker | 4373 (48.5%) | 320 (3.8) | 1.20 (1.01 to 1.42) | 1.24 (1.03 to 1.49) |
| Current Smoker | 1254 (13.9%) | 92 (3.9) | 1.21 (0.95 to 1.55) | 1.49 (1.16 to 1.93) |
| **Type of baseline MI** | | | | |
| STEMI | 4730 (52.4%) | 282 (3.1) | 1.00 (reference) | 1.00 (reference) |
| NSTEMI | 3764 (41.7%) | 301 (4.2) | 1.36 (1.15 to 1.60) | 1.14 (0.96 to 1.35) |
| Unknown | 533 (5.9%) | 38 (3.7) | 1.20 (0.85 to 1.68) | 1.15 (0.80 to 1.64) |
| **Type of management of index MI** | | | | |
| PCI | 7287 (80.7%) | 448 (3.2) | 1.00 (reference) | 1.00 (reference) |
| CABG | 664 (7.4%) | 38 (3.0) | 0.93 (0.67 to 1.30) | 0.81 (0.57 to 1.13) |
| Medical | 1076 (11.9%) | 135 (6.8) | 2.12 (1.75 to 2.57) | 1.78 (1.45 to 2.18) |
| **Years between index MI and enrolment** | | | | |
|  | 5326 (59.0%) | 372 (3.7) | 1.00 (reference) | 1.00 (reference) |
|  | 3701 (41.0%) | 249 (3.5) | 0.97 (0.82 to 1.13) | 1.00 (0.85 to 1.17) |
| **Category of diabetes** | | | | |
| No diabetes | 6009 (66.6%) | 341 (3.0) | 1.00 (reference) | 1.00 (reference) |
| Non-insulin-treated diabetes | 2235 (24.8%) | 170 (4.0) | 1.36 (1.13 to 1.63) | 1.44 (1.19 to 1.73) |
| Insulin-treated diabetes | 783 (8.7%) | 110 (7.7) | 2.60 (2.09 to 3.22) | 2.29 (1.83 to 2.86) |
| **Second prior MI** | | | | |
| No | 8103 (89.8%) | 506 (3.3) | 1.00 (reference) | 1.00 (reference) |
| Yes | 924 (10.2%) | 115 (6.7) | 2.06 (1.68 to 2.52) | 1.80 (1.46 to 2.21) |
| **Multi-vessel disease** | | | | |
| No | 3086 (34.2%) | 198 (3.4) | 1.00 (reference) | 1.00 (reference) |
| Yes | 5941 (65.8%) | 423 (3.7) | 1.11 (0.94 to 1.32) | 1.16 (0.97 to 1.38) |
| **Chronic kidney disease** | | | | |
| No | 8336 (92.3%) | 514 (3.2) | 1.00 (reference) | 1.00 (reference) |
| Yes | 691 (7.7%) | 107 (8.6) | 2.67 (2.17 to 3.29) | 2.15 (1.73 to 2.68) |
| **EQ5D overall score** | | | | |
| 0 | 4348 (48.4%) | 207 (2.5) | 1.00 (reference) | 1.00 (reference) |
| 1 | 1935 (21.6%) | 120 (3.2) | 1.32 (1.05 to 1.65) | 1.22 (0.97 to 1.53) |
| 2 | 1189 (13.2%) | 93 (4.1) | 1.67 (1.31 to 2.14) | 1.46 (1.14 to 1.88) |
| 3 | 702 (7.8%) | 78 (6.0) | 2.43 (1.88 to 3.16) | 1.96 (1.50 to 2.57) |
| 4-10 | 804 (9.0%) | 120 (8.3) | 3.39 (2.71 to 4.25) | 2.86 (2.25 to 3.63) |
| **EQ-5D mobility** | | | | |
| No problems | 6774 (75.4%) | 360 (2.8) | 1.00 (reference) | 1.00 (reference) |
| Some problems | 2192 (24.4%) | 255 (6.3) | 2.29 (1.95 to 2.69) | 1.87 (1.58 to 2.22) |
| Severe problems | 14 (0.2%) | 4 (18.2) | 6.62 (2.47 to 17.73) | 5.01 (1.85 to 13.58) |
| **EQ-5D self-care** | | | | |
| No problems | 8464 (94.3%) | 535 (3.3) | 1.00 (reference) | 1.00 (reference) |
| Some problems | 477 (5.3%) | 76 (8.9) | 2.69 (2.11 to 3.42) | 2.14 (1.67 to 2.75) |
| Severe problems | 39 (0.4%) | 8 (12.7) | 3.84 (1.91 to 7.72) | 3.18 (1.57 to 6.44) |
| **EQ-5D usual activities** | | | | |
| No problems | 7332 (81.7%) | 411 (2.9) | 1.00 (reference) | 1.00 (reference) |
| Some problems | 1545 (17.2%) | 183 (6.4) | 2.21 (1.85 to 2.63) | 1.89 (1.57 to 2.26) |
| Severe problems | 102 (1.1%) | 24 (14.5) | 4.99 (3.30 to 7.52) | 3.34 (2.19 to 5.09) |
| **EQ-5D pain** | | | | |
| No problems | 5763 (64.2%) | 298 (2.7) | 1.00 (reference) | 1.00 (reference) |
| Some problems | 2973 (33.1%) | 288 (5.2) | 1.93 (1.64 to 2.27) | 1.71 (1.45 to 2.03) |
| Severe problems | 244 (2.7%) | 33 (7.4) | 2.77 (1.94 to 3.97) | 2.24 (1.55 to 3.24) |
| **EQ-5D depression/anxiety** | | | | |
| No problems | 6937 (77.3%) | 453 (3.4) | 1.00 (reference) | 1.00 (reference) |
| Some problems | 1884 (21.0%) | 144 (4.0) | 1.19 (0.98 to 1.43) | 1.16 (0.96 to 1.41) |
| Severe problems | 158 (1.8%) | 22 (7.8) | 2.28 (1.49 to 3.50) | 2.27 (1.47 to 3.52) |
| **EQ5D visual analogue scale score (0-100)** | | | | |
| 92.5+ | 1063 (11.9%) | 47 (2.3) | 1.00 (reference) | 1.00 (reference) |
| 82.5-92.5 | 2062 (23.0%) | 116 (2.9) | 1.29 (0.92 to 1.80) | 1.31 (0.93 to 1.84) |
| 72.5-82.5 | 2644 (29.5%) | 158 (3.1) | 1.37 (0.99 to 1.89) | 1.31 (0.95 to 1.82) |
| 62.5-72.5 | 1475 (16.5%) | 114 (4.1) | 1.78 (1.27 to 2.51) | 1.70 (1.21 to 2.40) |
| 52.5-62.5 | 728 (8.1%) | 72 (5.3) | 2.32 (1.61 to 3.35) | 2.04 (1.41 to 2.97) |
| 42.5-52.5 | 604 (6.8%) | 72 (6.4) | 2.82 (1.95 to 4.07) | 2.26 (1.56 to 3.29) |
| 0-42.5 | 372 (4.2%) | 39 (5.7) | 2.50 (1.64 to 3.82) | 2.04 (1.32 to 3.15) |
| **Years in formal education** | | | | |
| No formal education | 357 (4.0%) | 26 (3.8) | 0.99 (0.66 to 1.49) | 0.88 (0.58 to 1.35) |
| 1-9 years | 2709 (30.4%) | 205 (4.0) | 1.04 (0.86 to 1.26) | 0.99 (0.81 to 1.22) |
| 10-12 years | 2817 (31.6%) | 205 (3.8) | 1.00 (reference) | 1.00 (reference) |
| 13-15 years | 1575 (17.7%) | 101 (3.4) | 0.88 (0.69 to 1.11) | 0.89 (0.70 to 1.13) |
| 16 years or more | 1463 (16.4%) | 77 (2.7) | 0.71 (0.55 to 0.93) | 0.72 (0.55 to 0.94) |
| **Cohabitation** | | | | |
| Living alone | 1209 (13.5%) | 92 (4.1) | 1.14 (0.91 to 1.42) | 1.01 (0.80 to 1.27) |
| Not living alone | 7726 (86.5%) | 525 (3.6) | 1.00 (reference) | 1.00 (reference) |
|  |  |  |  |  |
|  |  |  |  |  |
| **Chronic anemia** | | | | |
| No | 8769 (97.1%) | 578 (3.4) | 1.00 (reference) | 1.00 (reference) |
| Yes | 258 (2.9%) | 43 (9.4) | 2.73 (2.00 to 3.73) | 1.73 (1.25 to 2.39) |
| **Angina** | | | | |
| No | 8129 (90.1%) | 523 (3.4) | 1.00 (reference) | 1.00 (reference) |
| Yes | 898 (9.9%) | 98 (5.9) | 1.75 (1.41 to 2.17) | 1.43 (1.15 to 1.79) |
| **CHF** | | | | |
| No | 7994 (88.6%) | 478 (3.1) | 1.00 (reference) | 1.00 (reference) |
| Yes | 1033 (11.4%) | 143 (7.6) | 2.44 (2.02 to 2.94) | 1.85 (1.52 to 2.26) |
| **Cerebrovascular Revascularisation** | | | | |
| No | 8940 (99.0%) | 606 (3.6) | 1.00 (reference) | 1.00 (reference) |
| Yes | 87 (1.0%) | 15 (9.6) | 2.70 (1.61 to 4.50) | 1.84 (1.09 to 3.09) |
| **Atrial fibrillation** | | | | |
| No | 8300 (91.9%) | 547 (3.4) | 1.00 (reference) | 1.00 (reference) |
| Yes | 727 (8.1%) | 74 (5.5) | 1.58 (1.24 to 2.02) | 1.20 (0.93 to 1.54) |
| **Permanent pacemaker** | | | | |
| No | 8827 (97.8%) | 593 (3.5) | 1.00 (reference) | 1.00 (reference) |
| Yes | 200 (2.2%) | 28 (7.7) | 2.18 (1.49 to 3.19) | 1.53 (1.04 to 2.25) |
| **Valve replacement/repair** | | | | |
| No | 8928 (98.9%) | 615 (3.6) | 1.00 (reference) | 1.00 (reference) |
| Yes | 99 (1.1%) | 6 (3.2) | 0.88 (0.39 to 1.96) | 0.66 (0.29 to 1.47) |
| **Implantable Cardioverter Defibrillator** | | | | |
| No | 8834 (97.9%) | 600 (3.6) | 1.00 (reference) | 1.00 (reference) |
| Yes | 193 (2.1%) | 21 (5.8) | 1.63 (1.06 to 2.52) | 1.15 (0.74 to 1.79) |
| **Stroke** | | | | |
| No | 8625 (95.5%) | 573 (3.5) | 1.00 (reference) | 1.00 (reference) |
| Yes | 402 (4.5%) | 48 (6.4) | 1.84 (1.37 to 2.48) | 1.41 (1.05 to 1.90) |
| **TIA** | | | | |
| No | 8835 (97.9%) | 597 (3.5) | 1.00 (reference) | 1.00 (reference) |
| Yes | 192 (2.1%) | 24 (6.7) | 1.91 (1.27 to 2.87) | 1.40 (0.93 to 2.11) |
| **PVD** | | | | |
| No | 8426 (93.3%) | 534 (3.3) | 1.00 (reference) | 1.00 (reference) |
| Yes | 601 (6.7%) | 87 (8.0) | 2.40 (1.91 to 3.01) | 1.77 (1.40 to 2.24) |
| **Venous thrombo-embolism** | | | | |
| No | 8878 (98.3%) | 604 (3.6) | 1.00 (reference) | 1.00 (reference) |
| Yes | 149 (1.7%) | 17 (6.1) | 1.72 (1.06 to 2.79) | 1.45 (0.89 to 2.35) |
| **Major bleed** | | | | |
| No | 8774 (97.2%) | 580 (3.5) | 1.00 (reference) | 1.00 (reference) |
| Yes | 253 (2.8%) | 41 (8.9) | 2.57 (1.87 to 3.52) | 1.91 (1.38 to 2.64) |
| **Cancer** | | | | |
| No | 8417 (93.2%) | 560 (3.5) | 1.00 (reference) | 1.00 (reference) |
| Yes | 610 (6.8%) | 61 (5.3) | 1.53 (1.17 to 1.99) | 1.22 (0.93 to 1.60) |
| **COPD** | | | | |
| No | 8382 (92.9%) | 551 (3.4) | 1.00 (reference) | 1.00 (reference) |
| Yes | 645 (7.1%) | 70 (5.8) | 1.70 (1.32 to 2.18) | 1.45 (1.13 to 1.87) |
| **Esophageal Varices** | | | | |
| No | 9011 (99.8%) | 620 (3.6) | 1.00 (reference) | 1.00 (reference) |
| Yes | 16 (0.2%) | 1 (3.2) | 0.88 (0.12 to 6.28) | 0.54 (0.08 to 3.85) |
| **Liver disease** | | | | |
| No | 8993 (99.6%) | 618 (3.6) | 1.00 (reference) | 1.00 (reference) |
| Yes | 34 (0.4%) | 3 (4.7) | 1.29 (0.42 to 4.02) | 1.15 (0.37 to 3.59) |
| **Peptic ulcer** | | | | |
| No | 8733 (96.7%) | 592 (3.6) | 1.00 (reference) | 1.00 (reference) |
| Yes | 294 (3.3%) | 29 (5.2) | 1.47 (1.01 to 2.13) | 1.19 (0.82 to 1.74) |
| **Type of anti-thrombotic medication** | | | | |
| No APT | 471 (5.2%) | 52 (5.9) | 1.84 (1.38 to 2.46) | 1.63 (1.22 to 2.19) |
| SAPT | 6189 (68.6%) | 381 (3.2) | 1.00 (reference) | 1.00 (reference) |
| DAPT | 2367 (26.2%) | 188 (4.2) | 1.30 (1.09 to 1.55) | 1.36 (1.13 to 1.64) |
| **Optimal medical therapy (BB+ACE/ARB+LLD)** | | | | |
| No | 3803 (42.1%) | 258 (3.6) | 1.00 (reference) | 1.00 (reference) |
| Yes | 5224 (57.9%) | 363 (3.6) | 1.02 (0.87 to 1.20) | 0.98 (0.84 to 1.16) |
| **Any anticoagulant** | | | | |
| No | 8529 (94.5%) | 568 (3.5) | 1.00 (reference) | 1.00 (reference) |
| Yes | 498 (5.5%) | 53 (5.7) | 1.65 (1.24 to 2.18) | 1.31 (0.98 to 1.75) |
| **ACE inhibitor or ARB** | | | | |
| No | 2320 (25.7%) | 160 (3.6) | 1.00 (reference) | 1.00 (reference) |
| Yes | 6707 (74.3%) | 461 (3.6) | 0.99 (0.83 to 1.19) | 0.98 (0.81 to 1.18) |
| **Beta-blockers** | | | | |
| No | 1899 (21.0%) | 125 (3.4) | 1.00 (reference) | 1.00 (reference) |
| Yes | 7128 (79.0%) | 496 (3.7) | 1.06 (0.88 to 1.30) | 0.96 (0.79 to 1.18) |
| **Diuretics** | | | | |
| No | 6761 (74.9%) | 354 (2.7) | 1.00 (reference) | 1.00 (reference) |
| Yes | 2266 (25.1%) | 267 (6.4) | 2.34 (2.00 to 2.74) | 1.91 (1.62 to 2.26) |
| **Statin or other lipid-lowering drug** | | | | |
| No | 629 (7.0%) | 59 (5.0) | 1.00 (reference) | 1.00 (reference) |
| Yes | 8398 (93.0%) | 562 (3.5) | 0.70 (0.53 to 0.91) | 0.77 (0.59 to 1.01) |
| **Anti-depressants** | | | | |
| No | 8400 (93.1%) | 557 (3.5) | 1.00 (reference) | 1.00 (reference) |
| Yes | 627 (6.9%) | 64 (5.5) | 1.59 (1.23 to 2.06) | 1.41 (1.08 to 1.84) |
| **Cardiovascular event (last 6 months)** | | | | |
| No | 8592 (95.2%) | 559 (3.4) | 1.00 (reference) | 1.00 (reference) |
| Yes | 435 (4.8%) | 62 (7.9) | 2.32 (1.78 to 3.01) | 1.88 (1.44 to 2.47) |
| **Bleeding event requiring overnight hospital stay (last 6 months)** | | | | |
| No | 8960 (99.3%) | 608 (3.6) | 1.00 (reference) | 1.00 (reference) |
| Yes | 67 (0.7%) | 13 (10.8) | 3.02 (1.75 to 5.24) | 2.11 (1.21 to 3.69) |
| **Cardiac surgery (last 6 months)** | | | | |
| No | 8981 (99.5%) | 620 (3.6) | 1.00 (reference) | 1.00 (reference) |
| Yes | 46 (0.5%) | 1 (1.1) | 0.31 (0.04 to 2.22) | 0.25 (0.04 to 1.80) |
| **Non-cardiac surgery (last 6 months)** | | | | |
| No | 9008 (99.8%) | 618 (3.6) | 1.00 (reference) | 1.00 (reference) |
| Yes | 19 (0.2%) | 3 (8.8) | 2.45 (0.79 to 7.61) | 1.62 (0.52 to 5.06) |
| **Visit to cardiologist (last 6 months)** | | | | |
| No | 6147 (68.1%) | 397 (3.4) | 1.00 (reference) | 1.00 (reference) |
| Yes | 2880 (31.9%) | 224 (4.1) | 1.22 (1.03 to 1.43) | 1.07 (0.90 to 1.27) |
| **Hospital visit (last 6 months)** | | | | |
| No | 5431 (60.5%) | 334 (3.2) | 1.00 (reference) | 1.00 (reference) |
| Yes | 3547 (39.5%) | 284 (4.2) | 1.32 (1.13 to 1.55) | 1.15 (0.98 to 1.36) |
| **ER visit (last 6 months)** | | | | |
| No | 8514 (94.3%) | 553 (3.4) | 1.00 (reference) | 1.00 (reference) |
| Yes | 513 (5.7%) | 68 (7.2) | 2.12 (1.65 to 2.73) | 1.69 (1.30 to 2.19) |
| GP visit (last 6 months) |  |  |  |  |
| No | 7304 (80.9%) | 485 (3.5) | 1.00 (reference) | 1.00 (reference) |
| Yes | 1723 (19.1%) | 136 (4.2) | 1.20 (0.99 to 1.45) | 1.01 (0.83 to 1.24) |

*^a^djustment for age, sex, diabetes, chronic kidney disease, multi-vessel disease, and second prior MI at recruitment, region (fixed effect), and country (using random effect) using multivariable Poisson regression
^+^the primary composite outcome occurred in 621 patients over 2-years follow-up, comprising all-cause death (295 patients), MI (195 patients), unstable angina requiring revascularization (103 patients), and stroke (58 patients).

Abbreviations: ACE, angiotensin-converting enzyme; ARB, angiotensin receptor blockers; APT, antiplatelet; BB, beta-blockers; CABG, coronary artery bypass graft; CI, confidence interval; COPD, chronic obstructive pulmonary disease; DAPT, dual antiplatelet therapy; ER, emergency room; EQ-5D, EuroQol-5 dimensions; GP, general practitioner; MI, myocardial infarction; LLD, lipid-lowering drugs; NSTEMI, non-ST-elevation MI; PCI, percutaneous coronary intervention; SAPT, single antiplatelet therapy; STEMI, ST-elevation MI; TIA, transient ischemic attack.

eTable 2. Incidence of the primary composite endpoint by region and by country

|  | **Patients, n** | **No. with primary outcome  (rate per 100-person-years)** |
| --- | --- | --- |
| **Region** |  |  |
| Asia & Australia | 2815 | 165 (3.0) |
| Europe | 4126 | 284 (3.6) |
| Latin America | 1104 | 88 (4.2) |
| North America | 982 | 84 (4.6) |
| **Country** |  |  |
| Australia | 399 | 33 (4.3) |
| China | 750 | 55 (3.8) |
| India | 495 | 9 (0.9) |
| Japan | 692 | 43 (3.2) |
| South Korea | 479 | 25 (2.7) |
| Belgium | 141 | 13 (5.0) |
| Denmark | 300 | 10 (1.8) |
| Finland | 72 | 4 (2.8) |
| France | 37 | 4 (5.6) |
| Germany | 1048 | 92 (4.6) |
| Italy | 391 | 16 (2.1) |
| The Netherlands | 592 | 40 (3.6) |
| Norway | 46 | 0 (0.0) |
| Portugal | 91 | 4 (2.2) |
| Romania | 343 | 31 (4.9) |
| Spain | 623 | 45 (3.8) |
| Turkey | 177 | 13 (3.8) |
| UK | 265 | 12 (2.4) |
| Canada | 245 | 11 (2.3) |
| USA | 737 | 73 (5.4) |
| Argentina | 451 | 32 (3.7) |
| Brazil | 115 | 9 (4.1) |
| Colombia | 348 | 32 (4.9) |
| Mexico | 86 | 6 (3.7) |
| Venezuela | 104 | 9 (4.5) |

eTable 3. Comparison of TIGRIS and CONCORDANCE study populations

|  |  | TIGRIS | CONCORDANCE |
| --- | --- | --- | --- |
| **Follow up and outcome events** | | | |
| Primary composite outcome |  | 621/9027 (6.9%) | 296/4672 (6.3%) |
| Death |  | 295/9027 (3.3%) | 135/4672 (2.9%) |
| MI |  | 195/9027 (2.2%) | 121/4672 (2.6%) |
| Stroke |  | 58/9027 (0.6%) | 20/4672 (0.4%) |
| UA requiring revasc |  | 103/9027 (1.1%) | 50/4672 (1.1%) |
| Age over 65 |  | 5626/9027 (62.3%) | 2321/4672 (49.7%) |
| **Risk factors** | | | |
| Diabetes |  | 3018/9027 (33.4%) | 1158/4672 (24.8%) |
| 2nd prior MI |  | 924/9027 (10.2%) | 1068/4672 (22.9%) |
| Prior major bleed |  | 253/9027 (2.8%) | 71/4672 (1.5%) |
| PVD |  | 601/9027 (6.7%) | 240/4672 (5.1%) |
| CHF |  | 1033/9027 (11.4%) | 267/4672 (5.7%) |
| CKD |  | 691/9027 (7.7%) | 329/4672 (7.0%) |
| CV hospitalisation in last 6 months |  | 435/9027 (4.8%) | 899/4672 (19.2%) |
| Diuretic |  | 2266/9027 (25.1%) | 387/4672 (8.3%) |
| Medical management of index event |  | 1076/9027 (11.9%) | 1604/4672 (34.3%) |
| EQ5D category | 0-2 | 7472/8978 (83.2%) | 2444/3084 (79.2%) |
|  | 3 | 702/8978 (7.8%) | 230/3084 (7.5%) |
|  | 3 | 804/8978 (9.0%) | 410/3084 (13.3%) |

Abbreviations: CONCORDANCE, Australian Cooperative National Registry of Acute Coronary Care, Guideline Adherence and Clinical Events; CV, cardiovascular; EQ-5D, EuroQol-5 dimensions; MI, myocardial infarction. Numbers show n/N (Kaplan Meier %) for events and n/N (%) for risk factors

**eTable 4. Comparison of risk models for the primary CV outcome in TIGRIS and CONCORDANCE**

|  | **TIGRIS** | | **CONCORDANCE** | |
| --- | --- | --- | --- | --- |
|  | **Patients affected (%)** | **Risk ratio (95% CI)** | **Patients affected (%)** | **Risk ratio\ (95% CI)** |
| Age over 65 | 62.3 | 1.34 (1.12, 1.60) | 49.7 | 1.61 (1.22, 2.12) |
| Diabetes | 33.4 | 1.42 (1.20, 1.67) | 24.8 | 1.06 (0.80, 1.40) |
| 2^nd^ prior MI | 10.2 | 1.52 (1.24, 1.88) | 22.9 | 1.58 (1.19, 2.09) |
| CKD | 7.7 | 1.61 (1.29, 2.02) | 7.0 | 1.74 (1.20, 2.53) |
| CHF | 11.4 | 1.33 (1.08, 1.64) | 5.7 | 1.95 (1.33, 2.86) |
| PVD | 6.7 | 1.52 (1.20, 1.93) | 5.1 | 1.70 (1.13, 2.57) |
| CV event (prior 6 months) | 4.8 | 1.46 (1.11, 1.93) | 19.2 | 2.28 (1.74, 2.99) |
| Major bleed | 2.8 | 1.69 (1.22, 2.34) | 1.5 | 1.48 (0.66, 3.33) |
| Medical management of index event | 11.9 | 1.62 (1.33, 1.99) | 34.3 | 1.68 (1.29, 2.20) |
| On diuretic at enrolment | 25.1 | 1.62 (1.35, 1.93) | 8.3 | 1.09 (0.73, 1.63) |
|  |  |  |  |  |
| EQ-5D score of 3 | 7.8 | 1.47 (1.15, 1.88) | 7.5 | 1.24 (0.74, 2.07) |
| EQ-5D score ≥ 4 | 9.0 | 2.06 (1.67, 2.55) | 13.3 | 2.75 (1.93, 3.92) |

CHF, congestive heart failure; CKD, chronic kidney disease; CV, cardiovascular; MI, myocardial infarction; PVD, peripheral vascular disease.

eFigure 1. Distribution of risk index in the TIGRIS study population

**eFigure 2.** Model observed and predicted risk for the CV composite
